# Blueberry Counteracts BV-2 Microglia Morphological and Functional Switch after LPS Challenge

**DOI:** 10.3390/nu12061830

**Published:** 2020-06-19

**Authors:** Maria Giovanna De Caris, Maddalena Grieco, Elisa Maggi, Antonio Francioso, Federica Armeli, Luciana Mosca, Alessandro Pinto, Maria D’Erme, Patrizia Mancini, Rita Businaro

**Affiliations:** 1 Department of Experimental Medicine, Sapienza University of Rome, Viale Regina Elena 324, 00161 Rome, Italy; mariagiovanna.decaris@uniroma1.it (M.G.D.C.); alessandro.pinto@uniroma1.it (A.P.); 2 Department of Biochemical Sciences, Sapienza University of Rome, Piazzale Aldo Moro 5, 00185 Rome, Italy; maddalena.grieco@uniroma1.it (M.G.); antonio.francioso@uniroma1.it (A.F.); luciana.mosca@uniroma1.it (L.M.); maria.derme@uniroma1.it (M.D.); 3 Department of Medico-Surgical Sciences and Biotechnologies, Sapienza University of Rome, Corso della Repubblica 79, 04100 Latina, Italy; elisa.maggi@uniroma1.it (E.M.); federica.armeli@uniroma1.it (F.A.); rita.businaro@uniroma1.it (R.B.)

**Keywords:** blueberry, BV-2 cells, actin cytoskeleton, cell migration, Rho, Rac1, M1/M2 phenotypes, inflammatory cytokines

## Abstract

Microglia, the innate immune cells of the CNS, respond to brain injury by activating and modifying their morphology. Our study arises from the great interest that has been focused on blueberry (BB) for the antioxidant and pharmacological properties displayed by its components. We analyzed the influence of hydroalcoholic BB extract in resting or lipopolysaccharide (LPS)-stimulated microglia BV-2 cells. BB exerted a protective effect against LPS-induced cytotoxicity, as indicated by cell viability. BB was also able to influence the actin cytoskeleton organization, to recover the control phenotype after LPS insult, and also to reduce LPS-driven migration. We evaluated the activity of Rho and Rac1 GTPases, which regulate both actin cytoskeletal organization and migratory capacity. LPS caused an increase in Rac1 activity, which was counteracted by BB extract. Furthermore, we demonstrated that, in the presence of BB, mRNA expression of pro-inflammatory cytokines IL-1β, IL-6 and TNF-α decreased, as did the immunofluorescence signal of iNOS, whereas that of Arg-1 was increased. Taken together, our results show that, during the inflammatory response, BB extract shifts the M1 polarization towards the M2 phenotype through an actin cytoskeletal rearrangement. Based on that, we might consider BB as a nutraceutical with anti-inflammatory activities.

## 1. Introduction

Great interest has been focused on blueberry (BB) for the nutraceutical and pharmacological properties displayed by polyphenolic compounds isolated from its extracts. The cultivation of BB is spreading in many countries, not only because its taste is particularly pleasant to consumers, but also because there are increasing reports that demonstrate its health benefits through a wide range of mechanisms: antioxidant, anti-inflammatory, anti-bacterial [1,2]. Experimental evidence pointed out that a prolonged intake of fruit-derived polyphenols can provide an important support for the prevention and treatment of chronic multifactorial diseases such as diabetes, cardiovascular diseases, neurodegenerative diseases and cancer [1,2,3,4,5]. BB is part of the Ericaceae family, which includes the *Vaccinium genus,* represented by more than 400 species, characterized by fleshy fruits with high ascorbate and polyphenols levels. *Vaccinium genus* was shown to contain some of the highest levels of polyphenols compared to other plant species [6,7]. Polyphenolic compounds exert pleiotropic effects in favor of human health by counteracting oxidative stress formation and carrying out important anti-inflammatory activities. In this connection, their use may extend to all of the chronic diseases sustained by inflammatory processes, such as neurodegenerative diseases, cardiovascular and metabolic diseases, which, moreover, increase the risk of further developing comorbidities [8,9,10]. The powerful antioxidant activity of BB polyphenols, which has been extensively demonstrated, is especially exerted by polyphenol fractions rich in anthocyanins and phenolic acids [11,12,13,14].

Anthocyanins are potent antioxidants [15] and have the ability to prevent lipid oxidation [16] and scavenge free radicals [17]. Anthocyanins are able to scavenge reactive oxygen species (ROS) and modulate the activity of enzymes, such as xanthine oxidase-1 (XO-1) activity, superoxide dismutase (SOD) and heme oxygenase-1 (HO-1) [18].

Much interest has therefore focused on the compounds obtained from BB for their antioxidant and anti-inflammatory properties. Several studies have been conducted using in vitro cellular systems showing the ability of BB to inhibit the production of inflammatory mediators after stimulation with LPS [19], stress signaling [20] and NF-kB signaling [21]. These results were confirmed by several in vivo studies. Briefly, BB was shown to own anti-oxidant as well as anti-inflammatory properties, to induce neuroprotective mechanisms when supplemented to high fat diet fed mice [22,23,24,25], and to counteract acute inflammation in rats [26]. Several clinical trials have demonstrated the benefits of a blueberry-enriched diet. A polyphenol-rich extract from BB supplemented to elderly subjects for 6 months improved age-related episodic memory decline [27] and anthocyanin-rich BB provided cognitive benefits in older adults with cognitive complaints [28]. Moreover, BB was shown to exert immunomodulatory effects and attenuate oxidative stress and inflammation in obese adults or those affected by metabolic syndrome [29,30].

Regarding the phenolic acid role, several studies that were undertaken to identify the BB bioactive compounds responsible for both antioxidant and anti-inflammatory activities, revealed that, in the case of 16 BB samples, it has been possible to draw a correlation between antioxidant and anti-inflammatory activities, and the content of polyphenolic acids, in particular samples characterized by the greater anti-inflammatory capacities, matched the samples with the higher phenolic acid content [31]; moreover, chlorogenic acid was the most represented among the polyphenolic acids present in BB extracts [32]. BB phenolic acid mixture was found to show anti-inflammatory activity by inhibiting the nuclear factor-κB (NF-κB) activation and the production of such inflammatory cytokines as Tumor Necrosis Factor-α (TNF-α) and interleukin-6 (IL-6) induced by lipopolysaccharide (LPS) [33,34].

The main non-communicable chronic diseases are sustained by inflammatory processes involving cells of innate and adaptive immunity. The initiation, as well as the amplification, of the inflammatory processes mainly concerns the cells belonging to the monocyte–macrophage line that are recruited at the site of inflammation during the injury, and that in turn recruit other cellular elements by the release of chemokines and cytokines then contributing to the progression of the disease. During acute events, innate immunity cells release inflammatory mediators in order to eliminate the etiologic agents of the injury; at a later stage, these cells begin the repair, engulfing debris and activating a series of processes that will lead to healing. To perform these different functions, cells undergo peculiar phenotypic and functional polarizations that may amplify inflammation, sometimes leading to chronic processes or, conversely, promoting tissue remodeling to restore tissue homeostasis and resolve inflammation [35,36].

During the process of polarization toward a pro-inflammatory phenotype, a morphological modification takes place, involving a rearrangement of the cytoskeleton leading to the regulation of mobility and migration of the cells towards the inflammation site. Microglia, the brain’s innate immunity cells, become activated in neuroinflammatory condition leading to an increase in reactive oxygen and nitrogen species, and undergo several modifications according to their polarization state [37].

Therefore, microglia switch from a resting and ramified phenotype to an ameboid phenotype (M1) characterized by an enlarged body, devoid of ramifications favoring the migration over relatively long distances to accumulate at damage sites [38,39,40,41,42,43]. In this process, the key role is played by cytoskeleton, and, undoubtedly, F-actin represents the major contributor. Studies on the effects of BB on microglia morphological changes induced by inflammation are not well documented. The present paper aims at analyzing the effects exerted by BB (*Vaccinium corymbosum*)-derived phenolic compounds on murine microglia cells stimulated with LPS. We compared morphological changes of cells under LPS stimulation, in the presence or in the absence of the BB extract, and quantified random migration and chemotaxis in a scratch-wound assay and Boyden chambers in the same experimental conditions. Functional properties of cells undergoing different treatments were analyzed by measuring Rho GTPase proteins activity, pro-inflammatory cytokines gene expression by real time-PCR, and the expression of functional markers such as inducible nitric oxide synthase (iNOS) and Arginase-1 (Arg-1) by immunofluorescence analysis.

## 2. Materials and Methods

### 2.1. Materials and Chemicals

Blueberry *Vaccinium Corymbosum* (*Jewels cultivar*), LPS, 3-(4,5-dimethylthiazol-2-yl)-2,5 diphenyltetrazolium bromide (MTT), Trypan blue, 4′, 6-diamidino-2-phenylindole (DAPI) and rhodamine-conjugated phalloidin (TRITC-phalloidin) were purchased from Sigma-Aldrich (St. Louis, MO, USA). The primary antibodies iNOS and Arg-1 were from Cell Signaling Technology (Danvers, MA, USA), goat anti-rabbit TRITC secondary antibody was from Jackson ImmunoResearch (Europe Ltd., Cambridge House, St. Thomas’ Place, London, UK), goat anti-rabbit Alexa Fluor 488 secondary antibodies were from Biotium (Inc, Landing Parkway, Fremont, CA, USA). Culture medium, serum and Tripsin-EDTA 1X were from Aurogene (Rome, Italy). The miRNeasy Micro kit was obtained from QIAGEN (Hilden, Germany), NanoDrop One/One C and Active Rho and Rac Pull-Down and Detection Kit were from Thermo Fisher Scientific (Waltham, MA, USA). The High-Capacity cDNA Reverse Transcription kit and Power SYBR^®^ Green Master Mix was purchased from Applied Biosystems (Foster City, CA, USA).

### 2.2. Preparation of Blueberry Extract and LC-HR-MS^n^ Analysis

The BB extract was obtained from 2 g of freeze-dried blueberry extracted for 2 h at 25 °C with 30 mL of MeOH:H_2_O:CH_3_COOH (25:24:1) after extensive homogenization with a glass potter. The extract was then centrifuged at 1200× *g* for 5 min and filtered through a 0.2 µm filter, and the solution dried and lyophilized. For the chromatographic analysis, 10 mg of dried extract were dissolved in 1 mL of LC-MS grade MeOH and 2 µL was analyzed. LC-HR-MS/MS (High Resolution Tandem Mass Spectrometry) measurements were performed on a Dionex Ultimate 3000 UHPLC System, equipped with a quaternary pump, autosampler (100 µL sample loop, partial injection mode, 2 µL injection volume, sample temperature 8 °C), a photodiode array detector (PDA) (Thermo Fisher Scientific, Bremen, Germany). Chromatographic analyses were performed on a Waters C18 HSST3 column (100 mm × 1 mm i.d., 1.7 μm particle size). Solvent A was 0.1% aqueous HCOOH and solvent B was 0.1% HCOOH in CH_3_CN. The flow rate was 0.5 mL/min and column temperature was set at 25 °C. Elution was performed isocratically for the first minute with 2% B; from min 1 to min 6, solvent B was linearly increased to 55%; from 6 to 10 min, 20% A and 80% B; then, in 0.5 min, solvent B was set at 100% and maintained for 2 min. The column was re-equilibrated with 98% A and 2% B before the next injection. The effluent from the PDA detector was connected on-line to an LTQ-Orbitrap Elite mass spectrometer equipped with a high-temperature electrospray ionization (HESI) ion source, controlled by the Excalibur 2.7 software (Thermo Fisher Scientific, Bremen, Germany) and operated in the negative or positive ion mode. The ion spray voltage was set to 4.0 kV, sheath and auxiliary gases to 20 and 5 psi, respectively. The Orbitrap-MS spectra were acquired at the *m*/*z* range of 50–2000 with a resolution of 30,000. The tandem mass spectra were acquired by collision induced dissociation (CID) in a linear ion trap (LIT) at 35% normalized collision energy and isolation width of 2.0 *m*/*z*. The ions were detected at the FT-resolution of 30,000.

### 2.3. Cell Culture and Treatment

The BV-2 murine microglial cell line, kindly provided by Dr. Mangino, Sapienza University of Rome, was cultured in Dulbecco’s modified Eagle’s medium (DMEM), supplemented with 10% fetal bovine serum (FBS) and 1% penicillin-streptomycin, at 37 °C in a humidified incubator under 5% CO_2_, until they reached 90% confluence. The lyophilized BB extract was dissolved in dimethylsulfoxide (DMSO) (1 mg/mL) and serial dilutions were prepared in complete cell culture medium to treat cells, as specified below. Cells were plated at an appropriate density, according to each experimental setting, and treated with 10, 50 or 100 ng/mL BB extract in the presence or in the absence of 100 ng/mL LPS. Cells treated with only DMSO were used as control sample.

### 2.4. Cell Viability Assays

The cytotoxicity of the BB extract was assessed by MTT and Trypan blue assays. For the MTT test, BV-2 cells were seeded onto 96-well plates at a density of 3 × 10^3^ cells per well in 150 µL of complete medium, and incubated with 10, 50 or 100 ng/mL of BB extract in the presence or absence of 100 ng/mL of LPS for 24, 48 and 72 h. Tetrazolium salt 5 mg/mL, suspended in phosphate buffered saline (PBS), was added to cell cultures to a final concentration of 0.5 mg/mL and then incubated for 4 h. The formazan crystals were extracted from the cells with a solubilizing solution (DMSO). An ELISA reader was used to measure the optical density at a wavelength of 570 nm, reference length 630 nm. The results are shown as the percent viability of the treated groups relative to the control, which was considered 100%. For the Trypan blue exclusion test, 3 × 10^3^ BV-2 cells for each well were seeded onto 24-well plates and treated as above. After treatments, cells were detached with 1× Tripsin-EDTA, and 100 µL of cell suspension were mixed with 100 μL of Trypan blue solution for 3 min, and cell counts were performed using a hemocytometer under a light microscope (100 times magnification). The results are shown as the percent viability of the treated groups relative to the control, which was considered 100%.

### 2.5. Immunofluorescence Microscopy

For immunofluorescence analysis, 2 × 10^4^ BV-2 cells were grown on 12 mm coverslips in 1 mL of complete medium, stimulated with 10 ng/mL BB in the presence or not of 100 ng/mL LPS or DMSO as control, fixed in 4% paraformaldehyde for 30 min, followed by treatment with 0.1 M glycine in PBS for 20 min, and with 0.1% Triton X-100 in PBS for an additional 5 min to allow permeabilization. To analyze actin cytoskeletal organization, BV-2 cells were incubated with TRITC-phalloidin for 45 min. For detection of M1/M2 polarization markers, cells were incubated with primary antibodies raised against iNOS (diluted 1:100) or Arg-1 (diluted 1:50) and subsequently with goat anti-rabbit Alexa Fluor 488 or goat anti-rabbit TRITC secondary antibodies (diluted 1:100), finally marked with DAPI to highlight the nucleus. The fluorescence signal was analyzed using an Axio Observer inverted microscope, equipped with the ApoTome.2 System (Carl Zeiss Inc., Ober Kochen, Germany). The measurement of cell areas was carried out using Image J software and the values obtained were expressed in µm^2^.

### 2.6. Migration Assays

To evaluate cell migration, we employed two different techniques: Boyden transwell chambers and scratch migration assays. Boyden chamber was performed using transwell inserts with 8-μm pore size polycarbonate membrane (Costar, Corning, New York, NY, USA). A total of 5 × 10^4^ BV-2 cells, in 200 µL of complete medium, were added to the upper well. For the chemotaxis evaluation, 750 µL of complete medium, containing 10 ng/mL BB in the absence or presence of 100 ng/mL LPS or DMSO used as control, were added to the lower chamber. After 24 and 48 h of incubation, cells that migrated through the filter were stained with DAPI, to highlight the nuclei, and then counted under a microscope. For the scratch assay, 8 × 10^5^ BV-2 cells were seeded onto 35 mm plates and grown until confluence. Confluent monolayers were wounded with a sterile 100 μL pipette tip; after an intensive wash, the remaining cells were incubated with 10 ng/mL BB in the absence or presence of 100 ng/mL LPS or DMSO used as control. Migration into the open scar was documented after 24 h with microphotographs at different time points after wounding. The number of migrating cells was quantified by counting all cells within a 0.4 mm^2^ region inside each scratch using Image J software.

### 2.7. Pull Down Assay for Activated Rho GTPases

A pull down assay was performed using the “Active Rho Pull-Down and Detection Kit” (Thermo scientific—n°16116). Briefly, cells were plated at an appropriate density to have at least 500 μg of total protein to add to the column for each sample. The cells were scraped, collected in a tube and gently rinsed once in ice-cold tris-buffered saline (TBS) added with 1 mM phenylmethylsulfonyl fluoride (PMSF), proteases inhibitors cocktail and 1 mM Na_3_VO_4_. 10^6^ cells were lysed in 100 µL of Lysis/Binding/Wash Buffer (25 mM Tris HCl, pH 7.2, 150 mM NaCl, 5 mM MgCl_2_, 1% NP-40, 5% glycerol, proteases inhibitors cocktail, 1 mM PMSF and 1 mM Na_3_VO_4_), the lysates were incubated on ice for 15 min and centrifuged at 16,000× *g* for 15 min at 4 °C. To ensure the pull-down procedure, GTPγS and GDP, regarded as positive and negative control, were incubated with 500 μg of total protein, respectively. The samples were left at 30 °C for 15 min under constant stirring. The reaction was terminated by mixing the sample with MgCl_2_ at a final concentration of 60 mM on ice. The supernatant of each sample was passed through a column and incubated with 100 μL Glutathione Resin (50% slurry containing 0.05% sodium azide) and 400 μg of GST-Rhotekin-RBD (5–6 mg/mL) at 4 °C for 1 h with gentle rocking. The columns were washed three times with Lysis/Binding/Wash Buffer. Each washing step included an intermediate centrifuge at 6000× *g* for 10–30 s. The bound proteins were eluted with 2× SDS Sample Buffer (125 mM Tris HCl, pH 6.8, 2% glycerol, 4% sodium dodecyl sulfate (SDS) (*w*/*v*), 0.05% bromophenol blue and 5% β-mercaptoethanol). The samples were electrophoresed and analyzed by western blot with the anti-Rho antibody. The same procedure was carried out with a Rac1 pull down assay (Thermo scientific—n°16118).

### 2.8. Real-Time Quantitative PRC Analysis 

Total RNA was extracted from the control and treated BV-2 cells using the miRNeasy Micro kit (Qiagen, Hilden, Germany) and quantified using NanoDrop One/OneC (Thermo Fisher Scientific, Waltham, MA, USA). cDNA was generated using the High-Capacity cDNA Reverse Transcription kit (Applied Biosystem, Foster City, CA, USA). Quantitative real-time PCR (qPCR) was performed for each sample in triplicate on an Applied Biosystems 7900HT Fast Real-Time PCR System (Applied Biosystem, Cheshire, UK) through the program SDS2.1.1 (Applied Biosystem, Foster City, CA, USA) using the Power SYBR® Green PCR Master Mix (Applied Biosystem, Foster City, CA, USA). The primers for real-time PCR amplification were designed with UCSC GENOME BROWSER (http://genome.cse.ucsc.edu/; university of California, Santa Cruz, CA, USA) (Table 1). The primer pair sequences were matched by BLASTn to the genome sequence to identify the primer locations with respect to the exons. A comparative threshold cycle (C_T_) method was used to analyze the real-time PCR data, where the amount of target, normalized to the endogenous reference of β-Actin (ΔC_T_) and relative to the calibrator of untreated control (ΔΔC_T_), was calculated by equation 2 ^−ΔΔC^_T_ as previously described [44].

### 2.9. Statistical Analysis

Data were expressed as the mean values ± standard deviations (SD) from at least three independent experiments. Statistical analyses were performed using a one-way ANOVA analysis of variance with a post hoc Bonferroni multiple comparison test (GraphPad Software Inc., La Jolla, CA, USA). All results were considered statistically significant with *p* < 0.05.

## 3. Results

### 3.1. Major Compounds of BB Extract

**Figure 1:**
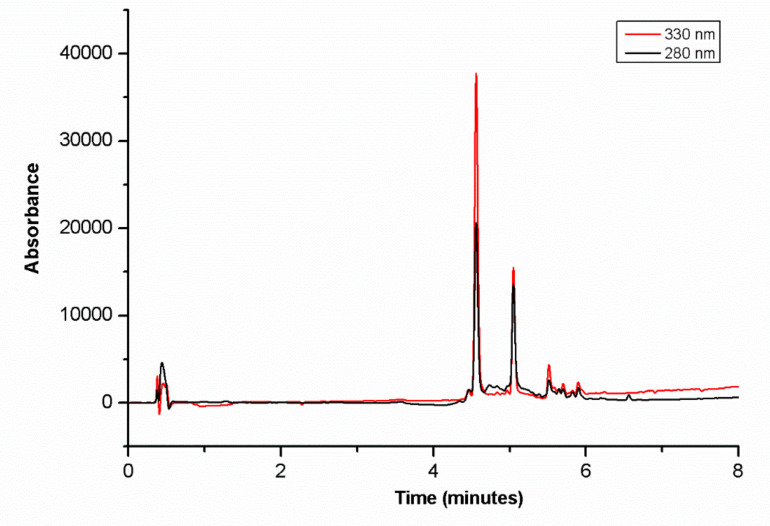
Chromatographic analysis of blueberry (BB) extract by UHPLC/MS/MS. The chromatogram was registered at 280 nm (black line) and 330 nm (red line).

To evaluate the composition of the BB extract, we performed a chromatographic analysis using UHPLC/MS/MS. The polyphenolic profile of the extract at 280 nm and 330 nm showed two major compounds, detectable with a retention time of 4.5 and 5 min (Figure 1).

The UV-vis spectra of both peaks suggest the presence of a caffeoyl moiety with a λ_max_ at 325 nm and a shoulder at 296 nm typical of conjugated hydroxycinnamic moieties. The MS spectrum showed, for both peaks, a pseudomolecular ion [M-H]^−^at 353.09 *m*/*z*, a [2M-H]^−^ ion at 707.18 *m*/*z* and an abundant fragment at 191.06 *m*/*z*. The founded ESIHRMS-molecular formula was C_16_H_17_O_9_^−^, corresponding to chlorogenic acid. MS/MS fragmentation of the 353.09 *m*/*z* precursor ion gives rise to 191 *m*/*z* product as the major fragment (C_7_H_11_O_6_^−^) corresponding to quinic acid and confirming the structure of both peaks as chlorogenic acid isomers [45,46,47,48,49,50]. Further characterization of the isomers structure was performed by using the method of Ncube et al. [51] and by comparison with chlorogenic acids of Virginia tobacco extracts. We analysed, according to the authors, Virginia tobacco extracts and compared the chromatographic and mass spectrometric fragmentation features of the different chlorogenic acids. Experimental results allowed us to identify the two isomers of our BB extract as trans- and cis- 5-Caffeoylquinic acid isomers, respectively. In addition to these two major constituents, another three minor compounds were detected in our extract. Compounds eluting at 5.42 and 5.61 min were identified as isomers of methyl caffeoylquinate, namely methyl 1-caffeoylquinate and methyl 4-caffeoylquinate, respectively. The founded ESIHRMS-experimental-molecular formula (C_17_H_19_O_9_^−^) and UV-absorbtion features (λ_max_ at 328 nm) of these isomers were in agreement with the proposed structures. ESI^-^MS/MS fragmentation of the molecular ion (3671 *m*/*z*) generates the caffeic acid base peak at 179.04 (secondary at 135.05 *m*/*z*) corresponding to the caffeic acid moiety, and the fragment at 191.06 *m*/*z* corresponding to the quinic moiety [52]. The last eluting (5.82 min) minor compound present in our extract was identified as delphinidin-rhamnoside. The founded ESIHRMS-molecular formula of this anthocyanin glicoside was C_21_H_19_O_11_^−^. ESI^-^MS/MS fragmentation of the molecular ion at 447.09 *m*/*z* generates a single ion at 301.04 *m*/*z* [53].

### 3.2. Influence of BB Extract on BV-2 Cell Viability

Before testing cell viability, we determined in BV-2 cells the amount of BB methanol extract by a dose–response curve with BB concentrations ranging from 10 ng/mL to 10 µm/mL. No cytotoxic effect was observed at lower concentrations, instead, the higher concentrations induced a slight reduction in vitality (data not shown). Based on these results, in the subsequent cell viability assays, BB extract was used at 10, 50 and 100 ng/mL. LPS was used at 100 ng/mL, as reported in the literature [19,54].

**Figure 2:**
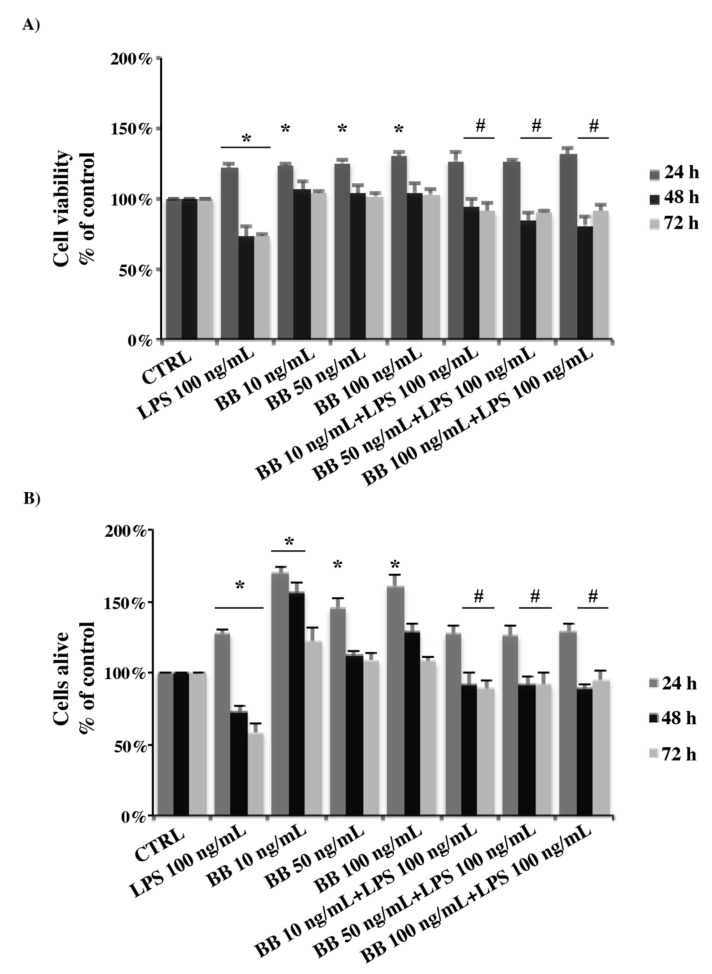
Analysis of cell viability evaluated by 3-(4,5-dimethylthiazol-2-yl)-2,5 diphenyltetrazolium bromide (MTT) assay (**A**) and Trypan blue exclusion test (**B**). Cells were treated with 10, 50 or 100 ng/mL BB extract in the absence or presence of 100 ng/mL lipopolysaccharide (LPS). Data were reported as percentage normalized to the control values and are mean ± SD of at least three independent experiments. * *p* < 0.05 compared to the same time points control, ^#^ *p* < 0.05 compared to the same time points LPS.

To detect the cytotoxic effect of BB extract on BV-2 murine microglial cells, we first performed the MTT assay. As shown in Figure 2A, treatment of microglial cells with BB alone for 48 h and 72 h at all the concentrations examined, did not basically change cell viability compared to the control cells, however, a slight increase in cell viability was evident at 24 h. LPS induced an increase in cellular viability after 24 h, and a significative reduction in viability at 48 h and 72 h of treatment (Figure 2A). The treatment of BB extract in combination with LPS was able to increase the cell viability compared to LPS-treated cells at 48 h and 72 h, although, at 24 h, a peak of activity was marked at all concentrations (Figure 2A). In addition, we performed a Trypan blue assay using cells treated as above, obtaining comparable results (Figure 2B). Thus, both assays have shown that, at none of the concentrations used, BB extract affected the cell viability of BV-2 cells in the presence of LPS. Therefore, subsequent experiments were performed using BB extract at a concentration of 10 ng/mL.

### 3.3. BB Extract and Cell Morphology Changes

**Figure 3:**
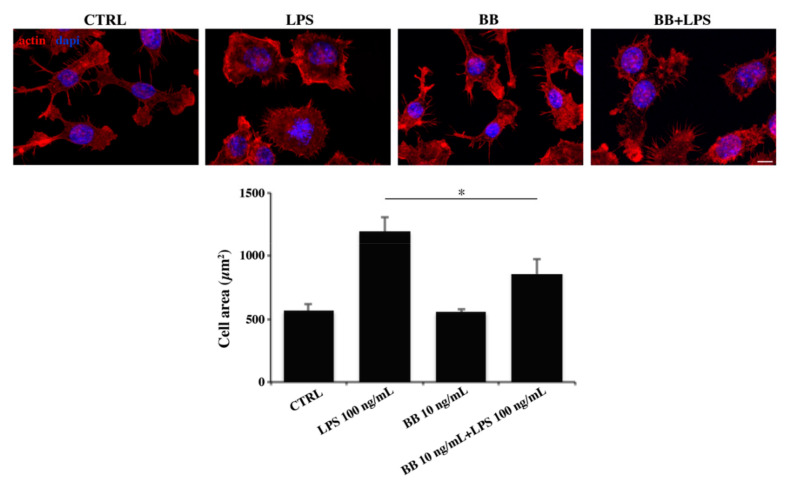
Analysis of cell morphology by rhodamine-conjugated phalloidin (TRITC-phalloidin) staining to highlight actin and 4’,6-diamidino-2-phenylindole (DAPI) to detect nucleus, after treatment with 10 ng/mL BB in the absence or presence of 100 ng/mL LPS. Bar: 20 µm. Cell areas were quantified using Image J software. * *p* < 0.05 compared to LPS.

Since microglia are able to modify their morphology in response to extracellular cues [55], we wondered whether BB was able to induce cellular changes in LPS-treated BV-2 cells. Hence, we performed immunofluorescence analysis using phalloidin staining of F-actin. Figure 3 shows that untreated cells presented the classic morphology of branched microglia, with a central soma and many elongated cellular processes. As expected, after LPS treatment, BV-2 cells increased their surface areas, as evidenced by the measure of the cellular areas reported in the graph (Figure 3), and acquired a polygonal morphology, with fewer and shorter branches. BB treatment did not affect cell morphology compared to the control, and in BB + LPS-treated cells, BB reverted LPS ameboid phenotype, with an increased ramification of distal branches (Figure 3).

### 3.4. Effect of BB Extract on Cellular Migration

**Figure 4:**
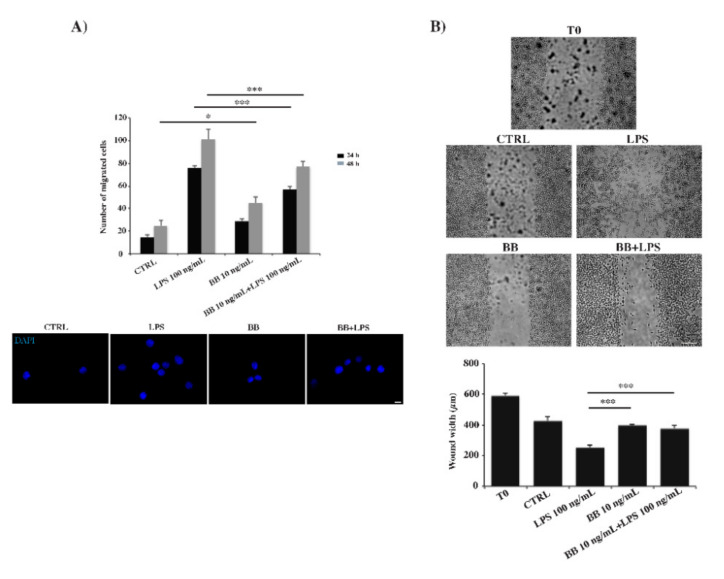
Analysis of cell migration determined by Boyden chamber assay (**A**) and scratch assay (**B**). Cells were treated with 10 ng/mL BB in the absence or presence of 100 ng/mL LPS. (**A**) Representative images of Boyden chamber assay using DAPI staining to mark the nucleus of cells migrated through the membranes. Bar: 200 µm. Data were reported as total number of cells migrated, randomly taken from twenty fields from three different experiments made in triplicate. (**B**) Representative images of scratch assay. Bar: 200 µm. Data were reported as mean of triplicate experiments ± SD. * *p* < 0.05 BB compared to control at 48 h, *** *p* < 0.001 compared to LPS.

To determine whether BB extract affects LPS-induced microglial motility, we used two different methods, Boyden transwell chambers and scratch migration assay. The chemotaxis experiment by Boyden assay showed that, after LPS treatment, a greater number of cells crossed the polycarbonate membrane compared to the control sample, both at 24 h and 48 h (Figure 4A). However, BB + LPS treatment was responsible for an intermediate number of migrated cells between LPS alone and BB, which alone induced a weak migration (Figure 4A). To examine the motility of BV-2 cells, we carried out a scratch assay. Microglial cells were allowed to migrate for 24 h in the scratch created in the monolayer, then fixed and free cell areas were measured. In Figure 4B, the results show that LPS induced a higher cell migratory potential compared to the control cells. Moreover, application of BB alone led to a slight motility, while, co-application of BB + LPS caused a significant reduction in microglial migration compared to LPS-treated BV-2 cells (Figure 4B). Thus, in both migration assays, BB acts as modulator reducing LPS-enhanced migration.

### 3.5. BB Extract Modulated Rho GTPases Activity

**Figure 5:**
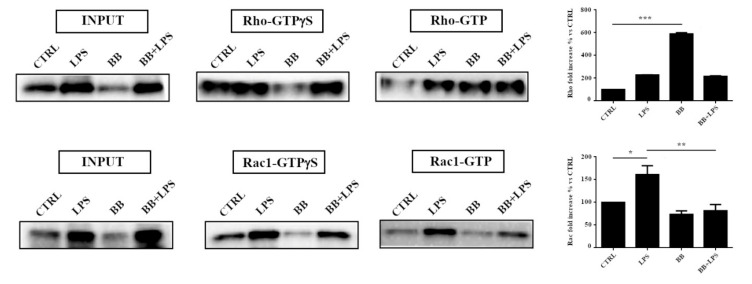
Pull down assay of Rho and Rac1 proteins. BV-2 cells treated with 10 ng/mL BB with or without LPS 100 ng/mL for 24 h. The pull down assay with GTPγS was used as positive control. The results were reported as a percentage versus control. Densitometric analyses were performed with ImageLab software (Biorad) and normalized to INPUT. INPUT was normalized on the β-actin signal. The data are the ± SD average of three independent experiments. *** *p* < 0.001 BB compared to control, * *p* < 0.05 LPS compared to control, ** *p* < 0.01 BB + LPS compared to LPS.

Since the Rho family proteins have a crucial role in the plasticity of the actin cytoskeleton, furthering morphological changes, we evaluated the influence of BB on this class of proteins by an active pull down assay for Rho and Rac1 proteins. This analysis allows the precipitation of only activated GTPase proteins, through a specific binding protein. Our results show an increase in activated Rho proteins in the BV-2 cells treated with BB compared to the control sample (Figure 5). We have also observed an increase in activated Rac1 proteins in the sample treated with LPS and a decrease in the sample treated with BB, in the presence or absence of LPS (Figure 5).

### 3.6. BB Extract Influenced Microglia M1/M2 Polarization Markers

**Figure 6:**
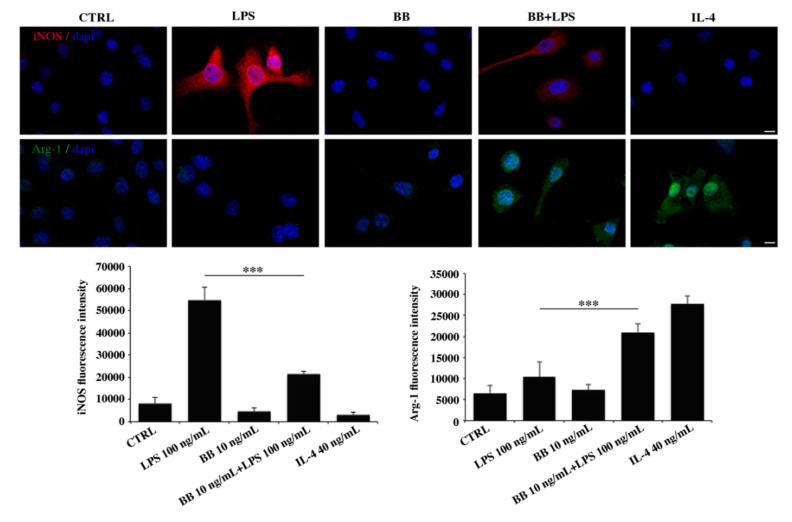
Immunofluorescence analysis of M1/M2 polarization markers. Cells were treated with 10 ng/mL BB in the absence or presence of 100 ng/mL LPS and stained for iNOS and Arg-1. IL-4 was used as positive control for Arg-1. Bar: 20 µm. Fluorescence intensity was quantified with Image J software. Results from three independent experiments are presented as mean ± SD. *** *p* < 0.001 compared to LPS.

To evaluate whether BB may induce a functional switch in LPS-treated BV-2 cells, we performed an immunofluorescence analysis assessing the expression of M1/M2 polarization markers. We observed that LPS significantly increased the expression of the M1 marker iNOS, whereas, adding to the BV-2 cultures IL-4, a well known polarization signal towards M2 phenotype, an increased expression of Arg-1 was detected (Figure 6). BB treatment alone did not exert any effect on the protein marker examined (Figure 6), conversely, if cells were treated with LPS in the presence of BB extract, we measured a decrease in iNOS expression together with an increase in Arg-1 (Figure 6). Taken together, these results indicate that BB is able to remodulate the M1 phenotype acquired by the cells after the insult with LPS towards the M2 phenotype.

### 3.7. BB Extract Prevented Pro-Inflammatory Cytokines Expression

**Figure 7:**
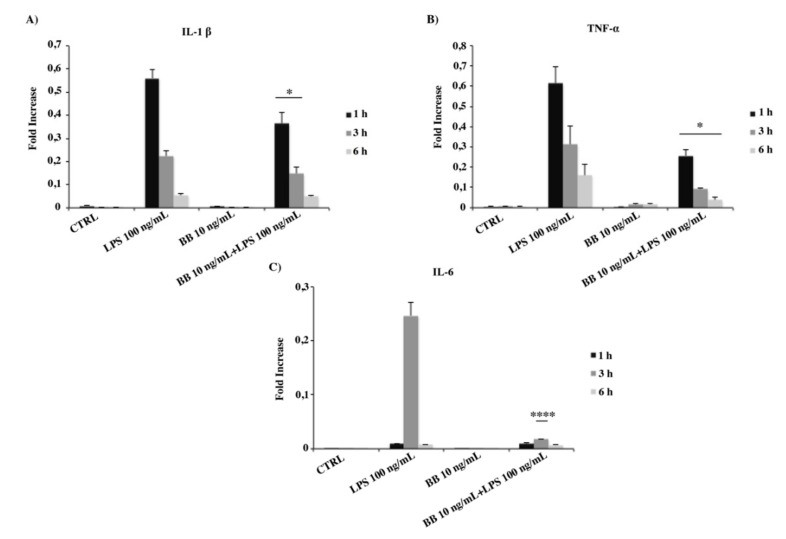
mRNA expression of IL-1 β (**A**), TNF-α (**B**) and IL-6 (**C**) monitored by qPCR and normalized to β-actin. Data are shown as mean ± SD from three independent experiments performed in triplicate. Expression profiles were determined using the 2^−ΔΔCT^ method. * *p* < 0.05 for IL-1β and TNF-α compared to LPS at the same time points, **** *p* < 0.0001 for IL-6 compared to LPS at the same time points.

To gain further insight into the potential anti-inflammatory role, the effect of BB extract on the mRNA expression of pro-inflammatory cytokines was assessed. In particular, we measured IL-1β, TNF-α and IL-6 mRNA expression levels. As showed in Figure 7, the expression of IL-1β and TNF-α on transcription level was the lowest in control and in BB-treated cells. When cells were induced with LPS, IL-1β and TNF-α, mRNA expression was sharply increased with the higher concentration at 1 h. In BB + LPS-treated cells, the expression of both cytokines was significantly decreased. In addition, the inhibitory effect of BB on IL-6 gene expression in LPS-treated cells is presented in Figure 7C. After stimulation with LPS, IL-6 mRNA expression showed the higher concentration at 3 h. When BB was added to media containing LPS, the IL-6 mRNA expression was significantly inhibited (Figure 7C).

## 4. Discussion

Several studies have highlighted the beneficial health effect of BB bioactive compounds such as anthocyanins, flavonols, pro-cyanidins and phenolic acids [56]. In particular, we focused our research on the BB *Vaccinium corymbosum, jewels cultivar,* whose chromatographic analyses revealed that the phenolic acids are the major compounds, whereas apparently, anthocyanins are not present in large amounts. This finding could be attributed to plant variety, ripening, cultivation practices, difference in temperature between day and night, growing area (central Italy instead of the Northern countries), processing and storage. In BB methanolic extract, the main polyphenol was chlorogenic acid, which is involved in several important and therapeutic roles as antioxidant, anti-inflammatory, neuroprotective, anti-microbial, and in the modulation of glucose and lipid metabolism [57]. Caffeoylquinate isomers and the delphinidin metabolite belonging to the anthocyanin glycoside family are also present in the extract. They are interesting compounds that are reported to increase ATP production, to prevent aggregation and neurotoxicity of the amyloid β-peptide in neuroblastoma cell lines and to inhibit immune checkpoints in human colorectal cancer cells [58,59,60]. The combination of these compounds makes BB extract an attractive material to explore for its possible use in dietary supplements. Considering the BB antioxidant and anti-inflammatory capabilities, we examined its putative protective effect against LPS-induced toxicity in microglia BV-2 cells. Our results suggest that the BB extract is able to modulate phenotypic and functional features of microglial cells. Indeed, treatment with LPS induced microglial proliferation at 24 h and subsequently reduced cell viability, which was restored by 10 ng/mL BB extract. The concentration of BB utilized in mouse/rat supplementation ranged from 200 mg/kg to 40 mg/kg or was up to 0.5 mg/mL when added to cell cultures, as reported by previous papers [22,23]. We showed that the low concentration utilized in our experimental conditions is already adequate to exert different cellular events, and represents a closer application as possible human nutraceutical supplementation.

Microglia cells rapidly respond to brain injury and disease by altering their morphology and phenotype to adopt an activated state. Microglia exist in various states of activation and retain the capability to shift their functional phenotype during the inflammatory response [61]. Firstly, these processes require actin cytoskeleton reorganization, which leads to several cellular responses, such as migration. Different compounds are present in the BB extracts obtained by Cui et al. (2019), and among them, quercetin, one of the most common flavonoids, was recently shown to interfere in macrophage migration processes induced by LPS. It significantly disrupted the F-actin cytoskeleton structure and promoted changes in the size and shape of the macrophages, affecting the formation of filopodia or lamellipodia, which are supported by actin filaments and associated proteins [62].

In our work, LPS stimulation caused a round ameboid shape in BV-2 cells, which is a sign of M1 polarization. BB extract in combined treatment with LPS was able to partially reverse this phenotype. This data are consistent with the study of Yorgun et al. (2017), showing that crocin treatment, a plant-derived carotenoid, reverses LPS ameboid phenotype in BV-2 microglial cells [63]. In addition, resveratrol, one the main polyphenols of grape, induces filopodia formation in a time- and concentration-dependent manner in MDA-MB-231 human breast cancer cells [64].

Dynamic rearrangement of the actin cytoskeleton is the first step in the microglial function of migration. Because BB extract modulated actin cytoskeletal morphology, we hypothesized that BB extract might affect the migration capability of BV-2 cells. Our results show that stimulation with LPS reliably elicited a robust migratory response, as also reported by Scheiblich et al. (2014) [65], and that BB extract in LPS-combined treatment significantly reduced microglial migration, both in scratch wound and Boyden chambers assays. As previously reported, the actin cytoskeleton remodeling associated with cell migration might be triggered via the activity of Rho family small GTPases [66,67,68]. Rho, Rac and Cdc42, components of the Rho GTPases-binding proteins family, coordinate many diverse cellular processes, such as cell polarity, migration, and cell membrane protrusion, however, one of the most relevant effects involves the rearrangement of the actin cytoskeleton during migration [69,70]. They are the key proteins for the proper neuronal development and survival [71]. Ridley and Hall (1992) and Nobes and Hall (1995), determined that activated Rho leads to the formation of stress fibers and focal adhesions, activated Rac promotes lamellipodia and membrane ruffles and activated Cdc42 promotes spike-like filopodia [72,73]. Hence, we observed an increase in activated Rac1 proteins in the sample treated with LPS and a decrease in the sample treated with BB in the presence or absence of LPS. Several studies demonstrated that the small GTPase Rac1 is a downstream effector for phosphatidylinositol 3-kinases (PI3-kinases), which affects the rearrangement of the actin cytoskeleton regulating actin polymerization in the lamellipodial protrusion [74,75]. It is well known that multiple signalling pathways are activated in response to LPS. Some of the pathways include the PI3-kinases/Akt [76,77,78]. As reported by Adams et al. (2010), the BB extract was able to inhibit the PI3K/AKT pathway in MDA-MB-231 cells, therefore, in our current work, we hypothesize that BB extract, added to LPS-treated cells, reduced BV-2 cell migration and actin reorganization through the modulation of the PI3K/AKT/Rac1 pathway [79].

Since actin cytoskeleton rearrangement influences different intracellular signaling pathways, as the production of inflammatory mediators, we investigated the effect of BB extract on the production of the M1/M2 polarization markers iNOS and Arg-1, and proinflammatory cytokines, including IL-1β, IL-6 and TNF-α. Our results suggest that BB extract is able to inhibit the expression of pro-inflammatory markers in BV-2 microglia cells stimulated by LPS. A decreased protein expression of iNOS, and a contemporary increased expression of Arg-1, were detected by immunofluorescence when cells were supplemented with BB in the presence of LPS, as expected, since L-arginine homeostasis in nonhepatic tissues, characterized by the competition between nitric oxide synthase (NOS) and arginase for the available intracellular substrate arginine, pushes microglia polarization toward the anti-inflammatory phenotype [80,81]. The polarization of arginine metabolism is driven by cytokines. T-helper (Th)1 cytokines (pro-inflammatory cytokines) induce iNOS, whereas Th2 cytokines (anti-inflammatory cytokines) up-regulate Arg-1 [82,83]. 

Surprisingly, we found that BB + LPS treatment results in higher levels of Arg-1 compared to the treatment with BB alone. LPS, as mentioned, is an inflammatory stimulus that polarizes cells belonging to the monocyte–macrophage series towards the phenotype M1 and, therefore, as expected, the levels of Arg-1, marker of M2 cells, are decreased in the presence of LPS. The more robust increase in Arg-1, after the stimulus with both BB and LPS, compared to the stimulus obtained with BB alone, deserves a comment. In 2008, Hirata et al. showed that LPS can induce an increase in inflammatory and anti-inflammatory cytokines as long as there is a synergy between TLR-4 ligands, such as LPS, and TLR-2 ligands [84], and possibly there is an interaction in their signalling pathways. Alternatively, in a way that is still unknown, BB extract could inhibit TLR-4 and consequently the activity of LPS, as described for other plant extracts [85]. 

Moreover, BB extract induces a down-regulation of IL-1β and TNF-α (pro-inflammatory cytokines) in LPS-stimulated microglia cells. Our results are in agreement with the studies of Figueira et al. (2017) showing that polyphenols prevent the transcription of inflammatory cytokines mRNAs by increasing the levels of IkBα, a protein that inhibits NF-kB translocation from the cytoplasm to the nucleus, blocking its ability to induce the transcription of pro-inflammatory genes [86]. In addition, a significant reduction in the production of TNF-α in LPS-stimulated RAW 264.7 cells by BB flavonoids is reported by Shi et al. (2017) [21]. Moreover, mRNA and protein levels of iNOS and cyclooxygenase-2 in LPS-activated BV-2 cells were significantly reduced by treatment with BB extract [18].

In a recent pilot study, we evaluated the postprandial effects of high fat high glycemic load meals, including BB, on five obese/overweight patients suffering from metabolic syndrome, and we showed that significant changes in cytokine gene expression levels occurred after meals with BB. In particular, the mRNAs expression of IL-6 and TGF-β, respectively, pro- and anti-inflammatory cytokines, significantly decreased and increased after BB supplementation, indicating a positive impact of BB intake in reducing the risk of inflammation [21].

## 5. Conclusions

Our results underline the homeostatic role of the BB extract in the presence of an inflammatory stimulus. Moreover, our data suggest an involvement of BB in actin cytoskeleton rearrangement, which affects migration processes and functional microglia polarization.

BB seems to be an important player in counteracting the inflammatory response by promoting the resting microglia phenotype acquisition. For all these issues, BB extract seems to have the potentiality and effectiveness to be utilized as a dietary supplement.

## Figures and Tables

**Table 1:** Primers used.

GENE	Forward Primer (5′–3′)	Reverse Primer (5′–3′)
mIL-1β	GAAATGCCACCTTTTGACAGTG	TGGATGCTCTCATCAGGACAG
mTNF-α	CTGAACTTCGGGGTGATCGG	GGCTTGTCACTCGAATTTTGAGA
mIL-6	CGGAGAGGAGACTTCACAGAGGA	TTTCCACGATTTCCCAGAGAACA
mACT-β	GGCTGTATTCCCCTCCATCG	CCAGTTGGTAACAATGCCATGT
